# Socioeconomic Status and Physical Activity among Mothers of Young Children in an Asian City: The Mediating Role of Household Activities and Domestic Help

**DOI:** 10.3390/ijerph17072498

**Published:** 2020-04-06

**Authors:** Alison Carver, Muhammad Akram, Anthony Barnett, Robin Mellecker, Ester Cerin

**Affiliations:** 1 Mary MacKillop Institute for Health Research, Australian Catholic University, Melbourne, VIC 3000, Australia; Muhammad.Akram@acu.edu.au (M.A.); Anthony.Barnett@acu.edu.au (A.B.); Ester.Cerin@acu.edu.au (E.C.); 2 Faculty of Education, The University of Hong Kong, Hong Kong, China; robmel@hku.hk; 3 School of Public Health, The University of Hong Kong, Hong Kong, China

**Keywords:** socioeconomic status, motherhood, family, household activities, mediation analysis, physical activity, Asia

## Abstract

Mothers of preschoolers (3 to 5 years old) risk being physically inactive. This study aimed to examine associations between socioeconomic status (education, employment, income) and moderate to vigorous physical activity (MVPA) among mothers of preschoolers in Hong Kong. Family functioning (e.g., having a domestic helper, division of household chores and child-related activities) was explored as a potential mediator of associations between socioeconomic indicators and the mother’s self-reported MVPA. Using zero-inflated negative binomial modelling confounder-adjusted associations between socioeconomic indicators and MVPA (total effects) were estimated. Mediation analyses (joint-significance test) were also performed. Using generalized linear mixed models, intermediate pathways were identified. No significant total effects of any socioeconomic indicator on the mother’s MVPA were found. However, mediation analyses identified a complex network of inconsistent mediators partly explaining their associations via eight pathways. Also, whilst non-residents/extended family playing with the child(ren) was not associated with any socioeconomic indicator, this was negatively associated with the mother’s MVPA. A further pathway was mediated by the mother playing with her child(ren). Extended family playing with the child(ren) was inversely associated with the mother doing so. Associations between socioeconomic indicators and MVPA among preschoolers’ mothers are complex and inconsistent, requiring further investigation in diverse contexts. Encouraging active play with their preschoolers may increase mothers’ physical activity levels.

## 1. Introduction 

The health benefits of regular physical activity across the lifespan are well-established. Compliance with global guidelines of 150 minutes per week of moderate- to vigorous-intensity physical activity is associated with reduced risks of overweight/obesity, cardiovascular disease, type 2 diabetes, and some cancers [1]. However, inequities exist regarding physical activity participation that are attributable to socio-demographic factors, such as gender, age, and socioeconomic status [1,2]. 

Overall, females tend to be less physically active than males [1], with differences being observed in childhood and adolescence [3] and continuing through adulthood [4,5]. One particular sub-group of females who may be particularly at risk of low physical activity levels comprises mothers of preschool children (aged 3–5 years). Whilst international studies indicate that total physical activity levels decrease linearly with age [4], leisure-time physical activity levels are shown to dip through the 20s and early 30s, bottoming out at around the age of 35 years before beginning to increase again [5]. These declines in physical activity may be due to work commitments during career establishment and setting up home (combined with financial responsibilities, e.g., mortgages), but also due to child-bearing and parenting of young children among young women in particular [6]. A growing body of research suggests that a woman’s transition to motherhood, specifically, is accompanied by a decline in her leisure-time physical activity levels [7]. Even among those mothers who enjoy being physically active, it may not always be easy to prioritise this over family responsibilities [8]. This challenge may be pertinent among Chinese mothers, in particular, due to cultural beliefs of collective familism, whereby family needs and obligations take precedence over individual interests and desires [9]. Yet, there is a paucity of research on factors that influence overall physical activity among mothers of young children [10], and none to our knowledge in an Asian urban setting.

A key factor that may impact on physical activity among mothers with young children is socioeconomic status. In many developed nations, the distribution of discretionary leisure-time physical activity, in particular, follows a socioeconomic gradient, whereby those who experience a greater advantage (e.g., more highly educated, higher income) are more likely to engage in habitual leisure-time physical activity than those who are more disadvantaged [2,11]. However, the effect of socioeconomic status on physical activity participation varies across cultures and ethnicities. In Hong Kong (China), for example, being educated beyond high school was inversely related to leisure-time physical activity, whilst positive associations were found in Western cities, such as Adelaide (Australia), Wellington (New Zealand), and Ghent (Belgium) [5]. In China, attitudes that value academic achievement over physical activity may generally be attributable in part to the cultural influence of Confucianism, a philosophy which measures worth and status by educational attainment, and places little value or emphasis on sporting pursuits or physical fitness [12]. In postcolonial Hong Kong, however, capitalist values that remain as a legacy of British rule promote further recognition of education and profession as indicators of status [13].

These influences of culture and ethnicity may also be reflected in the domains of physical activity in which Chinese adults engage and how these differ according to their socioeconomic status. For example, a study of adults in Jiaxing, China [14] found negative associations of total physical activity with educational attainment and individual-level income. These negative associations were mainly due to low-income and less educated individuals engaging in more occupational and household physical activity than their counterparts. In contrast, transportation and leisure-time physical activity tended to be positively related to education but unrelated to income. Also, although being unemployed was associated with more leisure time, transportation, and household physical activity than being employed, it resulted in lower overall physical activity, due to accruing only minimal amounts of or no occupational physical activity [14]. Given these findings, it is plausible to assume that aspects of socioeconomic status (educational attainment, income, and employment status) may also uniquely influence overall physical activity among Chinese mothers of young children by impacting on the time they need, or enabling them to invest in particular activity domains. For example, lack of cost-free childcare has been identified as an influential barrier to mothers’ engagement in leisure-time physical activity [15]. It is possible that some more advantaged households may be able to outsource childcare in order to free up the mother’s time to engage in her own employment (thus, providing some opportunity for the accumulation of occupational physical activity) and/or leisure-time physical activity. 

Another activity that may be outsourced by more advantaged households is housework, which is often cited as a barrier to leisure-time physical activity among mothers in general [16]. However, housework compounds this situation for those whose engagement in active leisure pursuits is already compromised due to caring for preschoolers [7]. On the other hand, housework and childcare may make a positive contribution to the accumulation of health-enhancing levels of physical activity for some mothers who are not interested in active recreational activities or participation in organized sport and do not enjoy being physically active. There is very limited research on family functioning including division of household chores and how this is related to total physical activity among mothers of preschoolers. The accumulation of health-enhancing levels of physical activity may be impacted by division of labour within the household, as well as the mother’s own employment patterns (i.e., whether she works full-time/part-time outside the home or is a full-time carer and homemaker) [16] and her self-efficacy regarding engagement in leisure-time physical activity [8]. 

A key strategy used by more advantaged families with young children in an attempt to reduce the burden of housework and childcare is to outsource these tasks through paid domestic/caring help [17]. One location where the hiring of domestic help is pervasive is Hong Kong [18]. According to the Legislative Council of Hong Kong [19], 11% of the 2.6 million domestic households in Hong Kong hire foreign domestic helpers, among whom almost all (98%) come from Indonesia or the Philippines. In the 1970s, the Hong Kong government introduced a policy to permit the hiring of these helpers to boost the local economy by freeing local married women from household duties and childcare to seek skilled employment. Married couples with children are the key employers of live-in foreign domestic workers, and among these nuclear families, the prevalence of this domestic arrangement increased from 13% to 30% between 1995 and 2016 [19]. 

**Figure 1:**
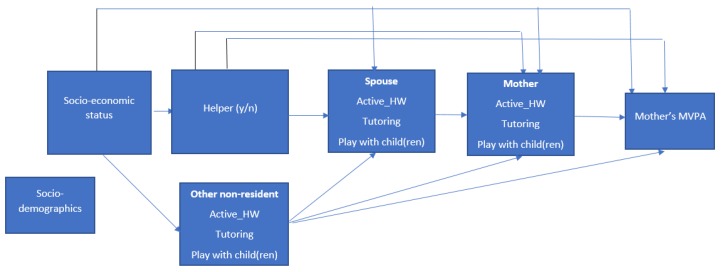
Conceptual model of socioeconomic status, household composition, activities, and their associations with the mother’s moderate to vigorous physical activity (MVPA)**.**

The current study examined whether and how socioeconomic status (operationalised here as mother’s educational attainment, her employment status, and household income) is associated with moderate to vigorous physical activity among mothers of preschoolers in Hong Kong. In particular, we aimed to develop and test a theoretical model of how family functioning (particularly the provision of a domestic helper, the division of household chores and childcare activities, including playing with children) mediates the associations between socioeconomic status indicators and total physical activity among mothers of preschoolers in Hong Kong (Figure 1). Specifically, we hypothesized that household income would be negatively related to overall physical activity because, in previous studies, it was associated with lower levels of occupational and household physical activity and unrelated to transportation and leisure activities [14]. Employment status was also expected to be negatively associated with overall physical activity because there is evidence that employed Chinese adults may accumulate less transportation, leisure, and household physical activity than their counterparts [14], and young working women are unlikely to compensate for the lack of participation in these physical activity domains with paid manual work [20]. 

Given that educational attainment is shown to be negatively related to occupational and household activities but positively associated with transportation and leisure physical activity [14], we hypothesised that it would be unrelated to overall physical activity. All associations between socioeconomic indicators and mother’s total physical activity were expected to, in part, be mediated by having a domestic helper and the mother’s participation in household tasks (Figure 1). Due to the lack of information on the impact of socioeconomic status on the division of housework within family members, no specific hypotheses were formulated on the mediating role of a partner’s and other family members’ participation in household tasks. The mother’s enjoyment of physical activity was also examined as a correlate and moderator, since it is shown to be positively associated with actual physical activity levels [21] and may determine the impact of the provision of domestic help on mothers’ physical activity levels. It was hypothesized that among mothers who receive more domestic help, those who enjoy physical activity may report higher levels of total physical activity (due to them being able to engage in leisure-time physical activity) and those who do not enjoy physical activity may report lower levels of total physical activity (due to them not engaging in any other form of physical activity).

## 2. Materials and Methods

We used data from a project on environmental and parental influences on Hong Kong preschoolers’ physical activity [22]. Chinese-speaking parents of preschoolers were recruited using convenience sampling between September 2011 and April 2014 in Hong Kong [22]. Ethical approval to conduct this study was obtained from the Human Research Ethics Committee for Non-Clinical Faculties of the University of Hong Kong (#EA560310). The recruitment process involved stratification by area-level socioeconomic status (median household income) and population density (based on Tertiary Planning Units (TPUs), the smallest administrative area units in Hong Kong with publicly available Census data) to maximize variability in these neighbourhood characteristics, which might influence household behaviours and engagement in various types of physical activity [22]. TPUs were categorised according to the median monthly value of household income of HK $24,500 as low-to-medium SES or medium-to-high SES. Similarly, regarding population density, TPUs were categorised as either low-density (≤9000 residents/km^2^) or high-density (>9000 residents/km^2^). This resulted in each TPU being classified into one of four strata: low-to-medium SES/high density (LSES/HD); low-to-medium SES/low density (LSES/LD); moderate-to-high SES/high density (HSES/HD); or moderate-to-high SES/low density (HSES/LD). The initial recruitment plan was to include around 100 parents per stratum. However, due to difficulties recruiting in medium-to-high SES areas, low SES areas were over-sampled to give a final sample that comprised 57 (14%; HSES/LD), 67 (16%; HSES/HD), 165 (40%; LSES/LD), and 122 (30%; LSES/HD) parents [22]. 

Parents (and primary carers) of at least one Chinese-speaking child aged 3–5 years attending kindergartens and Maternal and Child Health Centres in pre-selected areas of Hong Kong were approached by research assistants who invited them to participate in the study. Parents were ineligible to participate if they lacked Chinese reading and writing skills or if their child had a medical condition that affected their physical activity. Participants provided informed written consent prior to completing survey items on sociodemographic variables, perceived physical and social characteristics of their neighbourhood, as well as items on family functioning and behaviours. Participants completed the related questionnaires on-site or at home, returning these to the research team by reply-paid mail. 

### 2.1. Sample

Overall, 411 parents or caregivers (female, 79.8%; mean age 37.2 (SD 5.8) years) were recruited from Hong Kong kindergartens, as well as Maternal and Child Health Centres. The sample size of 411 participants was powered to detect a small effect size of approximately 2% of explained variance in the outcome of the original study [22]. Since mothers were the focus of the current study, the analytic sample comprises 322 mothers (and female primary carers). 

### 2.2. Outcome Variable 

Maternal physical activity was measured using the Chinese version of the International Physical Activity Questionnaire (short form), which has been shown to be reliable and valid for measuring total physical activity among Chinese samples [23]. Mothers reported on their physical activity during the week prior, particularly on the frequency (days) of and usual time spent (hours, minutes) in physical activity of moderate or vigorous intensity, and corresponding variables for walking. Total time (minutes) spent per week in moderate- to vigorous-intensity physical activity (MVPA) was derived by summing up the total time (no. of days multiplied by usual minutes per day) spent in physical activity of moderate and vigorous intensities, plus walking. Due to an excess of zero values for MVPA, two physical activity variables were created: “engagement in any MVPA” (yes/no) and “non-zero weekly time (minutes) of MVPA”. 

### 2.3. Exposure Variables

The following three variables were examined as indicators of the mother’s socioeconomic status: her education level (highest attained), employment status, and household income. Response options for education level ranged from 1 “uneducated” to 7 “post-graduate degree”. Employment status was reported using response options: 1, “not currently employed”, 2, “part-time”, 3, “full-time (40 h/week)” and 4, “more than 40 h/week”; then collapsed into three categories: 0, “unemployed”, 1, “part-time”, and 2, “full-time”. Household income (average monthly) was reported using 10 ordered categories, ranging from 1 “less than HKD 6000” to 10 “>HKD 59,999”. 

### 2.4. Mediators

Mothers survey-reported on the division of housework within their family. In particular, they were asked who performed the following 10 tasks: grocery shopping; cooking meals; laundry; house cleaning; repair work; taking care of parents when they need help; taking care of children when they are sick; tutoring children; playing with children; and shopping for other daily needs. Possible response options were: (1) “respondent”; (2) “spouse”; (3) “other resident”; (4) “parents living elsewhere”; (5) “children living elsewhere”; and (6) “other non-residents”. With reference to standardised criteria for categorising the International Classification of Activities for Time-Use Statistics (ICATUS) activity groups into sleep, sedentary behaviour, and physical activity [24], we classified all tasks as “active” (i.e., physical activity of light, moderate, or vigorous intensity), except for tutoring children (generally a sedentary behaviour) and playing with children, which may be active or sedentary (e.g., playing board games). We noted whether (yes/no) mothers tutored or played with their child(ren) and included these as separate variables (mediators) in analyses. 

For each mother, we computed the number of active housework tasks (maximum score: 8) they reported doing. Similarly, we computed the number of active tasks performed by their spouse and recorded whether (yes/no) they reported tutoring or playing their child(ren). We examined the tasks reported to be done by “parents living elsewhere”, “children living elsewhere”, and “other non-residents”. Combining these data, we derived scores for active housework tasks performed by non-residents (excluding non-resident domestic helpers) (possible range 0–24), as well as scores for tutoring (possible range 0–2 if we include adult tutors only) and for playing with children (possible range 0–3). We did not include variables representing the number of tasks performed by other residents because they were collinear with having a domestic helper (*r* = 0.71). 

To indicate the provision of paid domestic help, we derived a “helper” variable using data from two survey variables that indicated: (1) whether (yes/no) a helper lived in a household, and (2) whether a helper looked after the preschool child. There were cases where participants reported having a non-resident helper looking after their child (i.e., the helper comes to their home when necessary, but does not reside there). In some households there was a resident helper who did not have the task of looking after their child. The derived “helper” variable had three categories: 0 “No helper”; 1 “Non-resident”; and 2 “Resident”. 

### 2.5. Covariates and Moderators

The age of the mother (years) and number of children in the household were identified as covariates. Mothers reported on their level of agreement that they enjoyed physical activity using a five-point Likert scale, with response options ranging from 1 “strongly disagree” to 5 “strongly agree”. As noted earlier, this variable was included in analyses as a potential covariate because previous studies have demonstrated that enjoyment of physical activity is associated with overall physical activity levels [21] and a moderator of provision of domestic help and MVPA associations.

## 3. Data Analysis

Descriptive statistics were computed to summarise data. The summary statistics are reported as a percentage for each categorical variable and as a mean (standard deviation) for each continuous variable of interest. Our main outcome variable, MVPA, was zero inflated, therefore we used zero-inflated negative binomial (ZINB) modelling, accounting for TPU-level clustering, to estimate confounder-adjusted associations between socioeconomic indicators and MVPA among mothers of preschoolers (total effect of socioeconomic status on MVPA). Zero-inflated negative binomial models are used, in general, to model count data with excessive zeros compared to the number of zero expected according to a negative binomial distribution. 

Mediation analyses were performed in four steps (Figure 1). Step 1 examined whether having a non-resident or resident domestic helper was predicted by the mother’s indicators of socioeconomic status (educational attainment, employment status, and household income) adjusted for the number of children in the household and the mother’s age. Multinomial models adjusted for TPU-level clustering were used for this purpose. We also examined whether the mother’s socioeconomic status indicators predicted having non-residents (e.g., child’s grandparent) doing “active” housework, and/or tutoring and playing with the child(ren). Step 2 examined whether the mother’s socioeconomic status, having a helper and/or having another non-resident (e.g., child’s grandparent) who did “active” housework, tutoring, or played with the child(ren) predicted having a spouse who did “active” housework, tutoring, and played with their child(ren). Step 3 examined the associations of the mother’s socio-economic status, having a helper, the spouse’s and other non-residents’ active housework, tutoring, and playing with child(ren) with the mother’s active housework, tutoring, and playing with child(ren). Generalized linear mixed models (GLMM) with Poisson variance and logarithmic link functions (for number of active housework activities) or binomial variance and logit link functions (for tutoring and playing with child(ren)) were used to model these potential mediators (domestic help from non-residents in step 1; domestic help in steps 2 and 3). Step 4 examined the association of all variables included in the previous models, mother’s active housework, tutoring, and playing with child(ren) with mother’s MVPA. To identify significant mediators, we used the joint-significance test according to which a variable is considered a mediator if it is associated with the exposure (e.g., socioeconomic indicator) and with the exposure-adjusted outcome directly or via other mediators [25]. Finally, we estimated the moderating effect of the mother’s enjoyment of physical activity on the path between the mother’s active housework and MVPA, and on the path between having a helper and MVPA. All analyses were conducted in STATA 14.2 (StataCorp, 2015, College Station, TX, USA).

## 4. Results 

Our sample comprised 322 mothers whose demographics are presented in Table 1. Their mean age was 36.1 (s.d. 4.8) years, and on average they had 1 to 2 children. Forty percent had not completed high school, but over a third (37%) had attained a Bachelor’s or higher degree. A low proportion of mothers were employed part-time (10%) while the remainder were split almost evenly between those not engaged in paid employment (44%) and working full-time (46%). Just over half (55%) of households had no helper, while almost a quarter had either a non-resident childminder (23%) or a resident helper (22%). 

The distribution of MVPA among mothers was positively skewed, with a median value of 238 minutes per week and almost a fifth (18%) of mothers reporting no MVPA at all. Just over half (56%) reported that they enjoyed being physically active. 

Within the household, mothers tended to do more different types of active tasks when compared with their spouses. For example, around two thirds (66%) of mothers reported they undertook five or more different types of active tasks, while just over a fifth (21%) of their spouses did so. Compared with mothers, higher proportions of spouses performed no active tasks (1%, mothers; 18%, spouses) or only one type of task (4%, mothers; 28%, spouses). The distribution of active household tasks, tutoring, and playing with children (i.e., who did which tasks) is presented in Table 2. The only type of active task performed predominantly by spouses rather than by mothers was repair work; otherwise, higher proportions of mothers compared with spouses did each type of active household task. Active tasks performed by other non-residents tended to be done by the child(ren)’s grandparents, except for repair work which was undertaken by other non-residents. 

### 4.1. Indirect and Direct Effects of Socioeconomic Status on Mother’s MVPA 

**Figure 2:**
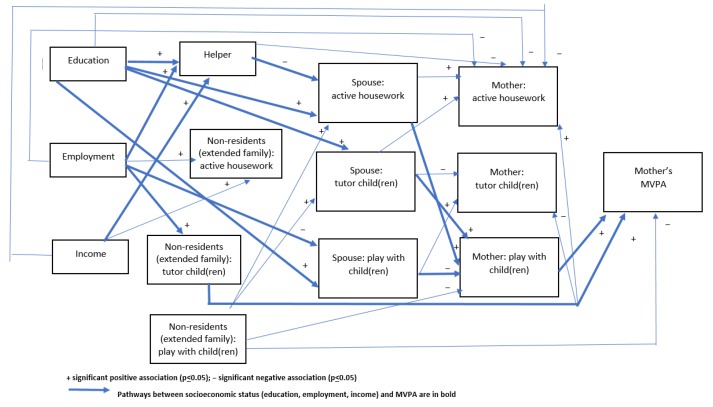
Direct and indirect effects of socioeconomic status on the mother’s moderate to vigorous physical activity.

No significant total effects of any component of socioeconomic status (i.e., education, employment, income) on the mother’s MVPA were found (Table 3). However, given that indirect (mediated) effects can exist in the absence of total significant effects [26], we conducted mediation analyses. Our mediation analyses identified a complex network of inconsistent mediators (i.e., with opposite effects) that partly explained the associations between these socioeconomic indicators and the mother’s MVPA (Figure 2) via eight pathways. In the first three pathways, the mother’s education, employment (full-time), and income were each positively associated with having a helper (Table 4) which mediated the pathway between each socioeconomic variable and her MVPA, via having her spouse doing active housework (inversely associated with having a helper) and the mother playing with her child(ren). The number of active household tasks performed by the spouse was positively associated with the mother playing with their child(ren) (Table 5) and this, in turn, was negatively associated with her reporting no physical activity (Table 6). A fourth mediating pathway existed that was similar to these, but included a direct positive association between the mother’s education and the spouse’s active housework (i.e., omitting the helper variable). Two additional pathways indicated that the mother’s education and employment (part-time) were associated positively and negatively, respectively, with her spouse playing with the child(ren) which, in turn, was negatively related to the mother playing with their child(ren) (Figure 2). The seventh pathway was represented by a positive indirect effect of the mother’s employment on her MVPA. This was mediated by having a non-resident (extended family) tutor her child(ren) (Figure 2). The eighth pathway indicated a positive effect of education on the mother’s MVPA via having the spouse tutoring the child(ren), and this having a positive effect on the mother playing with the child(ren) (Table 4 and Table 5; Figure 2). 

### 4.2. Patterns of Associations between Household Activity Division and Mother’s MVPA

Whilst having a non-resident (extended family) playing with the child(ren) was not associated with any socioeconomic status variable, this was directly (and negatively) associated with the mother’s MVPA. A further pathway between these variables was mediated by the mother playing with the child(ren). Extended family playing with the child was inversely associated with the mother doing so.

The mother’s active housework was inversely associated with all socioeconomic variables via having a helper, and also directly inversely associated with her education and income. In addition, her active housework was positively associated with that of her spouse, but there was no association with any active housework done by extended family. Mother’s tutoring was inversely associated with tutoring by her spouse and also by extended family. Her being employed full-time was associated with extended family doing tutoring, while her education level was positively associated with her spouse tutoring her child(ren). Mothers playing with their child(ren) was positively associated with her spouse’s active housework and tutoring, but negatively associated with her spouse and extended family playing with the child(ren). 

There was no moderating effect of the mother’s enjoyment of physical activity on the path between the mother’s active housework and MVPA, or on the path between having a helper and MVPA (data not shown).

## 5. Discussion

This study examined how socioeconomic status is associated with the physical activity of mothers of preschoolers in Hong Kong via its impact on the provision of domestic help and the distribution of household tasks. We found no total effect of socioeconomic status (i.e., education, employment, income) on the mother’s MVPA. However, overall mother’s socioeconomic status was inversely associated with her doing active housework. In support of this, a recent study [27] of the impact of hiring domestic helpers on women’s workforce participation reported that women of lower overall socioeconomic status but with upper high school education tended to hire domestic helpers so that they could work and contribute to the household income, while women of higher socioeconomic status hired domestic helpers to free up their time for leisure (which may not necessarily include physical activity). 

In our study, two indicators of socioeconomic status—the mother’s employment status and household income—were significantly associated with the hire of domestic (resident and non-resident) help, while educational attainment, a further indicator of socioeconomic status, was associated only with having a resident helper. Our finding that mothers who worked full-time had over four times higher odds of having a resident helper aligns with findings of another study in Hong Kong reporting that the mother’s contribution to household income (i.e., through her own employment) was a key predictor of the hiring of domestic help [18]. Compared with mothers who were not in paid employment or who engaged in part-time work, mothers who worked full-time were more likely to receive help from extended family to do active household tasks and tutoring of children. Mothers with higher, rather than lower income were also more likely to receive help from extended family with active household tasks—however, this had no association with MVPA among mothers. There were no significant associations between any socioeconomic variable and extended family playing with children. This may reflect traditional Chinese collectivistic culture [28], whereby inter-generational bonds are strong across society regardless of socioeconomic status. 

Two significant but inconsistent associations were found between help from extended family and mother’s MVPA—extended family tutoring her child(ren) was positively associated with mother’s MVPA, but extended family playing with her child(ren) was negatively associated with mother’s MVPA. It is possible that having extended family tutor children frees up time for the mother to engage in leisure-time physical activity. Why extended family playing with her child(ren) does not have a similar association with mother’s MVPA is unclear. One plausible explanation is that when extended family play with her child(ren), the mother does not do so. Despite the lack of an association between the mother playing with her child and the amount of time she spent in MVPA, she was more likely to report doing some MVPA if she played with her child. Future research should explore the type of activities and pursuits in which mothers and their children engage together to better understand the impact of these on the mother’s physical activity levels and to identify opportunities for intervention. For example, sedentary board games or screen-based leisure options could be replaced with active games and sports to increase the physical activity of the mother and the child. Further, a study of the mothers’ participation in MVPA with their preschoolers in Belgium [29] found that recreational walking and cycling with their children on weekends was significantly associated with MVPA among mothers and their children. 

Unexpectedly, we found that the mothers’ actual engagement in MVPA was not dependent on their own enjoyment of physical activity. This contrasts with findings of previous research that identified enjoyment of physical activity as an important predictor of habitual physical activity [30]. A possible explanation may be that Hong Kong mothers tend to accrue incidental physical activity while doing household chores or walking for transport rather than engaging in leisure-time physical activity. Promotion of active transport is a potential way for public health professionals to increase population levels of physical activity in subgroups who do not enjoy being physically active [3]. Urban planners can also play a role by designing activity-friendly urban environments [31].

The findings from mediation analyses shed further light on household dynamics. The mother’s participation in active housework was positively associated with her spouse also doing so (suggesting teamwork) and with her spouse and extended family tutoring her child(ren) (suggesting distribution of tasks). Our findings also suggest that distribution of some activities was associated with socioeconomic status. For example, more highly educated mothers had a helper and did fewer types of active household tasks compared to those with lower levels of education. The spouse’s involvement in active housework was associated with having a non-resident (mainly a grandparent) play with their child(ren), perhaps freeing up the spouse’s time for housework. Spouse’s involvement in active housework was inversely associated with having a non-resident childminder, but interestingly, was not associated with having a resident helper, even though previous research suggests an inverse association between these variables. For example, a qualitative study of working couples in Hong Kong suggested that husbands considered hiring domestic helpers as a “gift” to their wives, but this often resulted in husbands feeling absolved of their domestic responsibilities [32]. In our study, the outsourcing of domestic help and/or childminding appeared to reduce, but not preclude participation in active housework by mothers and spouses. A study in Hong Kong suggested that women often perceive the work done by their helper as sub-standard and, therefore, they supplement the household and caring duties even if they are in paid employment [32]. In addition, participation in household chores by mothers and spouses despite having a helper may be indicative of a high domestic workload. For example, a UK study of couples with children aged under 5 years that found no association between paid help and time spent by parents doing chores or caring for children, suggested that those who outsource domestic/caring help are overburdened [17].

Higher educational attainment among mothers was associated with greater involvement in active housework by spouses, and the association between mothers working full-time and spouses’ active housework also approached significance. These findings suggest that in households where the mother is more educated and has a career, there is less division of household labour according to more “traditional” roles where the male is the family breadwinner and the female is the homemaker and carer [18]. Whilst teamwork may have existed for active housework, tutoring of children and playing with them by mothers, spouses, and extended family appeared to be distributed tasks. For example, mothers were more likely to tutor their child(ren) if their spouse played with them, and were less likely to tutor if their spouse or a non-resident (grandparent) did so. Similarly, mothers were more likely to play with their child(ren) if their spouse did active housework or tutoring, and were less likely to play with them if their spouse or a grandparent did so. Spouses were more likely to play with their child(ren) when the mothers were more educated, but were less likely to do so if mothers worked part-time (and had more time to do this themselves) or if mothers reported playing with their child(ren). The involvement of grandparents in playing with and tutoring children may be related to strong family connections and filial piety in Chinese culture [33]. In addition, collective familism promotes family members striving together towards family prosperity and success [9]. 

Playing together as a family was not apparent from our results. A study of Chinese parents and their play with toddler children (i.e., slightly younger than our sample) identified that mothers and fathers engaged in different amounts and types of play with their children, and this varied according to the child’s sex [34]. For example, overall, mothers played more with their children, but mothers tended to play social games with daughters while fathers tended to engage in physical play with sons [34]. Stratification of analysis by sex of the child in the current study was not appropriate due to the small sample size and the fact that more than 60% of the sample had two or more children in a household of different ages. 

Even though mothers playing with their children was not significantly associated with the amount of MVPA accrued by the mother, playing with her child(ren) was positively associated with recording some MVPA. Encouraging mothers to engage in physically active play with their children may be an important intervention target for promoting physical activity among mothers of preschoolers. From a public health perspective, kindergartens and Maternal and Child Health Centres could provide recommendations for active play among mothers and children and offer opportunities for this in the local neighbourhood. Our measure of playing with children did not record whether this involved active or sedentary pursuits (a limitation of our study). Had this measure recorded only active play, a significant association with mother’s MVPA may have been observed. Despite the median amount of MVPA accrued by mothers in our study exceeding recommended levels, there is still a need for intervention, given that almost a fifth reported doing no physical activity. Also, their physical activity was self-reported, and thus it may have been prone to over-reporting [35]. It would have been interesting to include a retrospective measure of physical activity levels at earlier life stages (e.g., adolescence, pre-pregnancy) to explore whether those who were more physically active than their peers prior to motherhood remained so when their child was a preschooler. Further limitations were the cross-sectional study design with convenience sampling of a specific population and context that preclude, respectively, causal inference and generalisability of findings to populations other than mothers of young children in other highly urbanised settings in Asia. Strengths included stratification of the sample by area-level socioeconomic status and population density enabling the inclusion of participants living in diverse neighbourhood environments. 

## 6. Conclusions

Potential pathways of influence of socioeconomic indicators on MVPA among mothers of preschoolers are complex and inconsistent, and therefore require further investigation in a variety of contexts. Our findings suggest that encouraging mothers to engage in active play with their preschool children may increase mothers’ overall physical activity levels. Kindergartens, as well as maternal and child healthcare centres could play a role in promoting this through the provision of information and/or opportunities for active play among mothers and preschoolers.

## Figures and Tables

**Table 1:** Sample characteristics (N = 322).

	Mean (sd) or %
Mother’s age (years)	36.1 (4.8)
Education (%)	
Lower secondary or below	40.1
Higher secondary/diploma	22.7
Bachelor’s degree or above	37.3
Employment	
Not working	44.4
Part-time	9.6
Full-time	46.0
Household income	
<HKD 10,000	7.5
HKD 10,000–29,999	43.8
>HKD 30,000	48.7
Mother enjoys PA (% agree)	55.5
No. of children	1.73 (0.69)
MVPA (total minutes/week)	383.7 (427.8)
No MVPA (0 minutes/week, %)	17.7
Helper	
No helper	54.5
Non-resident, childminding	23.4
Resident	22.1
No. of active household task types	
done by mother	5.1 (1.9)
done by spouse	2.6 (2.3)
done by other non-residents (extended family)	0.9 (1.7)

**Table 2:** Division of housework in family.

Activity	Mother N (%)	Spouse N (%)	Other Resident N (%)	Parents Living Elsewhere N (%)	Children Living Elsewhere N (%)	Other Non-Resident N (%)
Grocery Shopping	270 (84.4)	113 (35.3)	72 (22.5)	31 (9.7)	0	4 (1.3)
Cooking meals	186 (58.1)	67 (20.9)	123 (38.4)	40 (12.5)	0	7 (2.2)
Laundry	207 (64.7)	64 (20.1)	125 (39.1)	14 (4.4)	1 (0.3)	7 (2.2)
Cleaning your house	209 (65.3)	71 (22.2)	126 (39.4)	16 (5.0)	1 (0.3)	9 (2.8)
Repair work	74 (23.1)	228 (71.3)	32 (10.0)	15 (4.7)	0	30 (9.4)
Take care of parents	128 (40.1)	69 (21.6)	30 (9.4)	56 (17.5)	1 (0.3)	8 (2.5)
Take care of children	282 (88.1)	103 (32.2)	66 (20.6)	29 (9.1)	0	2 (0.6)
Tutor children	309 (96.6)	150 (46.9)	42 (13.1)	30 (9.4)	1 (0.3)	4 (1.3)
Play with children	285 (89.1)	187 (58.4)	58 (18.1)	36 (11.3)	8 (2.5)	3 (0.9)
Shopping for other daily needs	278 (86.9)	114 (35.6)	68 (21.3)	23 (7.2)	0	4 (1.3)

**Table 3:** Associations of mothers’ socio-economic status indicators with moderate to vigorous physical activity (total effects ^a^).

	Non-Zero Minutes/Week of MVPA		Odds of Reporting 0 Minutes/Week of MVPA	
	e*^b^* (95% CI)	*p*	OR (95% CI)	*p*
**Education**	1.00 (0.86,1.16)	0.988	0.71 (0.48,1.05)	0.088
**Employment**				
(ref: none)				
Part time	1.00 (0.71,1.40)	0.979	1.05 (0.40,2.77)	0.914
Full time	0.81 (0.62,1.04)	0.102	1.31 (0.72,2.37)	0.381
**Income**	0.97 (0.93,1.02)	0.212	1.08 (0.93,1.25)	0.324

Notes: Results from zero-inflated negative binomial regression models adjusted for mother’s age and no. of child(ren) in the household. Income and education were modelled as continuous variables (i.e., linear terms) because this yielded a substantially better model fit than when the same variables were treated as categorical. MVPA = moderate to vigorous physical activity; e*^b^* = exponentiated value of regression coefficient; OR = odds ratio; CI = confidence intervals; *p* = *p*-value; ref = reference category. ^a^ These models provide information on the total effects of socio-economic indicators on mothers’ MVPA.

**Table 4:** Associations of mothers’ socio-economic status indicators with the presence of a domestic helper and household activities undertaken by other non-residents/extended family members ^a^ (Step 1 of mediation analysis).

		Presence of Domestic Helper (ref: None)			Other Non-Resident/Extended Family Members					
	Non-Resident		Resident		No. of Active Household Activities		Tutoring Children (ref: No)		Playing with Children (ref: No)	
	OR (95% CI)	*p*	OR (95% CI)	*p*	e*^b^* (95% CI)	*p*	OR (95% CI)	*p*	OR (95% CI)	*p*
**Education**	1.12 (0.84,1.50)	0.437	**1.42 (1.03,1.94)**	**0.030**	0.90 (0.77,1.05)	0.166	1.15 (0.87,1.52)	0.341	1.06 (0.83,1.34)	0.656
**Employment**										
(**ref: none**)										
Part time	0.38 (0.12,1.25)	0.113	1.02 (0.35,3.01)	0.967	0.97 (0.37,2.50)	0.944	2.36 (0.57,9.75)	0.235	1.06 (0.29,3.92)	0.931 0.072
Full time	**2.23 (1.09,4.57)**	**0.029**	**4.11 (1.61,10.5)**	**0.003**	**2.02 (1.22,3.35)**	**0.006**	**3.54 (1.46,8.57)**	**0.005**	1.87 (0.95,3.69)	
**Income**	**1.53 (1.18,1.98)**	**0.001**	**1.41 (1.10,1.82)**	**0.007**	**1.17 (1.07,1.28)**	**0.001**	1.13 (0.91,1.41)	0.252	1.18 (0.99,1.40)	0.069
**Enjoy PA**	0.87 (0.65,1.17)	0.355	**0.70 (0.52,0.95)**	**0.024**	**1.21 (1.04,1.41)**	**0.011**	1.57 (0.97,2.54)	0.069	1.26 (0.88,1.81)	0.210

Notes. Results from multinomial regression models (outcome: Presence of helper) and generalized linear mixed models with Poisson variance and logarithmic link functions (outcome: number of active household activities) or binomial variance and logistic link functions (outcomes: tutoring children and playing with children) adjusted for mother’s age and no. of child(ren) in the household. Income and education were modelled as continuous variables (i.e., linear terms) because this yielded a substantially better model fit than when the same variables were treated as categorical. e*^b^* = exponentiated value of regression coefficient; OR = odds ratio; CI = confidence intervals; *p* = *p*-value; ref = reference category; PA = physical activity. ^a^ These models provide information on the effects of socio-economic indicators on four potential mediators (Presence of domestic helper; Other non-resident/extended family members undertaking three types of household activities) of the associations of mothers’ socio-economic status with their moderate to vigorous physical activity (Step 1 of mediation analysis). Bold denotes *p* < 0.05.

**Table 5:** Associations of mothers’ socio-economic status indicators, presence of a domestic helper and household activities undertaken by other non-residents/extended family members with household activities undertaken by spouses and mothers ^a^ (Steps 2 and 3 of mediation analysis).

			Spouse						Mother			
	No. of Active Household Activities		Tutoring Children (ref: No)		Playing with Children (ref: No)		No. of Active Household Activities		Tutoring Children (ref: No)		Playing with Children (ref: No)	
	e*^b^* (95% CI)	*p*	OR (95% CI)	*p*	OR (95% CI)	*p*	e*^b^* (95% CI)	*p*	OR (95% CI)	*p*	OR (95% CI)	*p*
**Presence of domestic helper (ref: none)**												
Non-resident	**0.70 (0.56,0.87)**	**0.001**	1.11 (0.53,2.33)	0.776	1.26 (0.64,2.46)	0.503	**0.84 (0.73,0.96)**	**0.012**	0.56 (0.09,3.58)	0.539	1.77 (0.52,5.98)	0.358
Resident	0.96 (0.74,1.24)	0.740	1.33 (0.60,2.94)	0.481	1.50 (0.73,3.09)	0.271	**0.87 (0.78,0.99)**	**0.027**	1.44 (1.08,19.3)	0.781	2.34 (0.57,9.58)	0.239
**Education**	**1.13 (1.04,1.22)**	**0.002**	**1.40 (1.11,1.76)**	**0.004**	**1.28 (1.05,1.57)**	**0.017**	**0.96 (0.94,0.99)**	**0.010**	1.01 (0.54,1.89)	0.985	1.14 (0.76,1.70)	0.536
**Employment** (**ref: none**)												
Part time	0.73 (0.51,1.07)	0.104	0.41 (0.15,1.17)	0.097	**0.39 (0.16,0.92)**	**0.031**	0.99 (0.88,1.12)	0.885	NA ( 0 count in a cell)		1.07 (0.25,4.57)	0.927
Full time	1.25 (0.99,1.57)	0.058	1.73 (0.92,3.23)	0.087	1.17 (0.67,2.04)	0.586	**0.82 (0.76,0.89)**	**<0.001**	0.36 (0.06,2.32)	0.282	0.51 (0.18,1.45)	0.208
**Income**	0.98 (0.94,1.02)	0.330	0.94 (0.80,1.10)	0.422	0.95 (0.83,1.09)	0.462	**0.98 (0.96,1.00)**	**0.018**	1.18 (0.81,1.72)	0.392	1.02 (0.81,1.28)	0.877
**Enjoy PA**	1.00 (0.90,1.10)	0.922	0.89 (0.66,1.21)	0.471	0.83 (0.63,1.09)	0.182	1.02 (0.98,1.07)	0.285	0.67 (0.27,1.64)	0.379	1.24 (0.77,1.99)	0.370
**ONR/EF**												
No. of active household activities	0.98 (0.89,1.07)	0.592	0.95 (0.77,1.17)	0.605	1.01 (0.83,1.22)	0.952	0.98 (0.95,1.00)	0.060	1.14 (0.74,1.74)	0.552	1.37 (0.95,1.97)	0.091
Play with child (ref: no)	**1.23 (1.01,1.51)**	**0.043**	**3.63 (1.08,12.2)**	**0.037**	1.45 (0.53,3.93)	0.471	0.99 (0.88,1.11)	0.813	3.24 (0.32,32.8)	0.321	**0.16 (0.04,0.68)**	0.013
Tutor child (ref: no)	1.31 (0.88,1.94)	0.180	1.58 (0.33,7.69)	0.569	2.94 (0.66,13.0)	0.156	**1.23 (1.07,1.41)**	**0.004**	**0.03 (0.00,0.67)**	**0.027**	2.28 (0.33,15.8)	0.404
**Spouse**												
No. of active household activities							**1.05 (1.02,1.07)**	**<0.001**	1.29 (0.82,2.04)	0.278	**1.98 (1.41,2.80)**	**<0.001**
Play with child (ref: no)							0.97 (0.85,1.10)	0.632	**38.5 (3.46,429)**	**0.003**	**0.08 (0.03,0.24)**	**<0.001**
Tutor child (ref: no)							**1.13 (1.01,1.27)**	**0.030**	**0.05 (0.00,0.52)**	**0.013**	**7.69 (2.49,23.8)**	**<0.001**

Notes. Results from generalized linear mixed models with Poisson variance and logarithmic link functions (outcome: number of active household activities) or binomial variance and logistic link functions (outcomes: tutoring children and playing with children) adjusted for mother’s age and no. of child(ren) in the household. Income and education were modelled as continuous variables (i.e., linear terms) because this yielded a substantially better model fit than when the same variables were treated as categorical. e*^b^* = exponentiated value of regression coefficient; OR = odds ratio; CI = confidence intervals; *p* = p-value; ref = reference category; PA = physical activity; ONR/EF = other non-residents/extended family. ^a^ These models provide information on the effects of socio-economic indicators on potential mediators (Spouse and mother undertaking three types of household activities) of the associations of mothers’ socio-economic status with their moderate to vigorous physical activity (Steps 2 and 3 of mediation analysis). Bold denotes *p* < 0.05.

**Table 6:** Associations of mothers’ socio-economic status indicators and potential mediators with mothers’ moderate to vigorous physical activity ^a^ (Step 4 of mediation analysis).

	Non-Zero Min/Week of MVPA		Odds of Reporting 0 Min/Week of MVPA	
	e*^b^* (95% CI)	*p*	OR (95% CI)	*p*
**Presence of domestic helper**				
**(ref: none)**				
Non-resident	1.09 (0.79,1.50)	0.591	1.43 (0.66,3.13)	0.367
Resident	0.73 (0.51,1.04)	0.084	1.26 (0.57,2.78)	0.560
**Age**	0.98 (0.95,1.00)	0.107	0.99 (0.91,1.07)	0.768
**Education**	1.04 (0.91,1.19)	0.578	0.72 (0.46,1.11)	0.133
**Employment**				
(ref: none)				
Part time	0.90 (0.62,1.30)	0.559	0.93 (0.28,3.06)	0.901
Full time	0.87 (0.67,1.12)	0.274	1.28 (0.62,2.65)	0.507
**Income**	0.98 (0.93,1.04)	0.593	1.07 (0.93,1.23)	0.374
**Enjoy PA**	1.04 (0.91,1.18)	0.563	0.72 (0.51,1.02)	0.062
**ONR/EF**				
No. of active household activities	0.99 (0.93,1.07)	0.861	1.14 (0.92,1.42)	0.228
Play with child (ref: no)	**0.56 (0.34,0.92)**	**0.022**	0.43 (0.05,4.08)	0.463
Tutor-child (ref: no)	**1.98 (1.01,3.90)**	**0.047**	1.60 (0.14,19.0)	0.709
**Spouse**				
No. of active household activities	1.02 (0.96,1.09)	0.529	0.86 (0.73,1.02)	0.083
Play with child (ref: no)	0.91 (0.66,1.27)	0.592	0.98 (0.32,3.03)	0.970
Tutor-child (ref: no)	0.84 (0.60,1.17)	0.302	1.93 (0.69,5.42)	0.210
**Mother**				
No. of active household activities	1.05 (0.96,1.15)	0.319	1.00 (0.82,1.22)	0.988
Play with child (ref: no)	0.81 (0.52,1.27)	0.360	**0.19 (0.06,0.56)**	**0.003**
Tutor-child (ref: no)	1.60 (0.56,4.58)	0.382	2.32 (0.33,16.2)	0.397

Notes. Results from zero-inflated negative binomial regression models adjusted for mother’s age and no. of child(ren) in the household. Income and education were modelled as continuous variables (i.e., linear terms) because this yielded a substantially better model fit than when the same variables were treated as categorical. e*^b^* = exponentiated value of regression coefficient; OR = odds ratio; CI = confidence intervals; *p* = *p*-value; ref = reference category; PA = physical activity; ONR/EF = other non-residents/extended family; MVPA = moderate to vigorous physical activity. ^a^ These models provide information on the direct effects of socio-economic indicators on mothers’ MVPA and the associations of potential mediators (Presence of a domestic helper; Other non-resident/extended family members, spouse and mother undertaking three types of household activities) with mother’s MVPA (Steps 4 of mediation analysis). Bold denotes *p* < 0.05.
